# Isolated Small Fiber Neuropathy and Chemotherapy-Associated Cognitive Dysfunction Following Treatment for Gastric Adenocarcinoma: A Case Report of a Unique Entity

**DOI:** 10.7759/cureus.94412

**Published:** 2025-10-12

**Authors:** Haneen A Salama, Muhammad Umair, Isabella R Rojas, Peter G Bernad

**Affiliations:** 1 Faculty of Medicine, University of Benghazi, Benghazi, LBY; 2 Faculty of Medicine, Jinnah Sindh Medical University, Karachi, PAK; 3 Faculty of Medicine, ICESI University, Cali, COL; 4 Neurology, Neurology Services, Inc, Washington D.C., USA

**Keywords:** c-fiber dysfunction, chemobrain, chemotherapy-induced neurotoxicity, emg-negative neuropathy, flot, gastric adenocarcinoma, sfn, small fiber neuropathy, sudoscan

## Abstract

Small fiber neuropathy (SFN) is a poorly documented complication in oncology patients and is often overlooked due to a lack of awareness and testing. Concurrent chemotherapy-induced cognitive impairment (“chemobrain”) adds diagnostic complexity. We present a rare case of a 44-year-old male who developed SFN with intact large fibers, along with progressive cognitive slowing after completing neoadjuvant and adjuvant chemotherapy for gastric adenocarcinoma. Despite normal nerve conduction and brain MRI, the patient’s autonomic testing displayed severely abnormal sudomotor and microcirculatory responses. This report underscores the need for awareness of selective small fiber damage and neurotoxicity in cancer survivors while highlighting the diagnostic value of autonomic and sweat-based evaluations in unexplained peripheral symptoms post-chemotherapy.

## Introduction

Neurotoxicity related to chemotherapy manifests primarily as a length-dependent, sensorimotor polyneuropathy involving large fibers. However, selective small fiber involvement may occur independently and often escapes detection by conventional electrophysiological methods such as electromyography (EMG) and nerve conduction studies (NCS) [1,2]. Small fibers are responsible for temperature sensation, pain, and autonomic regulation, and their dysfunction can cause disabling symptoms like burning, numbness, orthostatic hypotension, and dysautonomia [3]. Chemotherapy can also result in central nervous system-related side effects, including attention deficits, executive dysfunction, and slowed processing speed, which is a phenomenon known as “chemobrain” [4,5]. In addition to its central effects, chemotherapy-induced neurotoxicity often affects the peripheral nervous system, particularly large fibers, leading to numbness, tingling, or motor dysfunction. However, a growing body of evidence indicates that small fibers may also be selectively affected, potentially through mechanisms such as oxidative stress, mitochondrial dysfunction, and inflammatory cytokine cascades [6,7]. We present a rare case of isolated small fiber neuropathy (SFN) with concurrent cognitive decline following FLOT (5-fluorouracil, leucovorin, oxaliplatin, and docetaxel) chemotherapy for the treatment of gastric adenocarcinoma despite normal EMG and MRI findings.

## Case presentation

A 44-year-old right-handed male network engineer was diagnosed with gastric adenocarcinoma (T2 N0, HER2-negative) involving the lesser curvature of the stomach. He underwent four cycles of neoadjuvant FLOT chemotherapy, followed by robotic subtotal gastrectomy with Billroth II and Braun anastomosis. Postoperatively, he completed four additional cycles of adjuvant FLOT. Surgical pathology confirmed a poorly differentiated adenocarcinoma invading the muscularis propria, with negative margins and lymph node involvement. Following chemotherapy, the patient reported persistent fatigue, daytime sleepiness, and cognitive slowing, accompanied by marked difficulty focusing. He experienced frequent band-like headaches and episodes of lightheadedness upon standing. Additionally, he developed tingling, numbness, and cold sensations in his hands and feet. These symptoms were disabling and significantly interfered with his daily occupational functioning.

Neurological examination revealed normal cranial nerve function, strength, speech, and gait. EMG and NCS were normal for both upper and lower extremities, and an MRI of the brain was unremarkable. However, a Sudoscan indicated severely impaired sudomotor function in the feet, while TM Flow testing confirmed abnormal C-fiber responses, with values of 435 in both the left and right foot. Nitric oxide microcirculatory peaks were also severely reduced, measuring 435 on the left and 422 on the right. Cardiac autonomic testing indicated abnormal parasympathetic function, as evidenced by a Valsalva ratio of 1.14 and a K30/15 ratio of 0.98 (Table 1). These findings supported a diagnosis of isolated SFN with autonomic involvement.

**Table 1:** Summary of relevant tests and lab work Abnormal values are presented in boldface EMG: electromyography; NCS: nerve conduction studies; MRI: magnetic resonance imaging

Test	Result	Normal range
EMG and NCS (upper and lower extremities)	Normal	
MRI of the brain	Unremarkable	
Sudoscan	Severely impaired sudomotor function in the feet	
TM Flow testing	Abnormal C-fiber responses (left and right foot showing values of 435)	Normal values typically >700
Nitric oxide microcirculatory peaks	Severely reduced (435 on the left and 422 on the right)	Normal >1000 mV
Cardiac autonomic testing - Valsalva ratio	1.14	>1.21
Cardiac autonomic testing - K30/15 ratio	0.98	>1.04
Sleep study	Conducted to rule out obstructive sleep apnea	
Thyroid	Within normal limits	
B12	Within normal limits	
Folate	Within normal limits	
Glucose	Within normal limits	
Vitamin D	38.9 ng/mL	>30 ng/mL

A sleep study was conducted to rule out obstructive sleep apnea. The patient’s daytime sleepiness persisted despite a trial of modafinil, which he discontinued due to side effects. Laboratory investigations, including thyroid function tests, vitamin B12, folate, and glucose levels, were within normal limits, although vitamin D was marginally low at 38.9 ng/mL. The patient had no history of alcohol or tobacco use and reported intermittent use of over-the-counter protein shakes and testosterone injections for muscle mass support.

No large fiber involvement was evident clinically or electrophysiologically, and cognitive complaints persisted months after chemotherapy, consistent with post-chemotherapy cognitive impairment.

## Discussion

This report highlights two under-recognized but significant complications of chemotherapy: selective SFN and chemotherapy-associated cognitive impairment. FLOT, particularly due to oxaliplatin and docetaxel, is known to be neurotoxic [6]. Neurotoxicity is a major dose-limiting complication of many chemotherapeutic agents and can present across a wide neuroanatomical spectrum, from peripheral axons to central neural networks. Platinum-based agents such as oxaliplatin and taxanes like docetaxel may disrupt microtubule transport, impair mitochondrial respiration, and trigger immune-mediated neuronal injury. Although large fiber damage is classically described, selective SFN can occur independently and is often missed by routine EMG and NCS [7,8]. Emerging studies suggest that chemotherapeutic agents can alter cytokine profiles and blood-brain barrier integrity, contributing to both peripheral neuropathy and central cognitive dysfunction.

While large fiber involvement is commonly reported, selective small fiber damage may occur early in the course or as an isolated phenomenon [7,8]. Such neuropathies are characterized by burning, tingling, and autonomic disturbances but often yield normal EMG and NCS results [3,9]. Despite normal electrophysiological testing in our patient, sweat-based and microvascular autonomic assessments confirmed SFN, supporting emerging evidence on diagnostic approaches [10]. The patient also demonstrated cognitive slowing and poor concentration, consistent with chemotherapy-related cognitive dysfunction (“chemobrain”). This complication is increasingly recognized in cancer survivors and is known to involve neuroinflammation, oxidative stress, blood-brain barrier disruption, and white matter changes - even in the absence of structural abnormalities on MRI [4,5,11,12]. Studies suggest that docetaxel and fluorouracil may contribute to central neurotoxicity by disrupting blood-brain barrier integrity and neuronal plasticity [5,12].

Notably, SFN has also been reported in the context of cancer itself, either as a paraneoplastic phenomenon or secondary to nutritional deficiencies, autoimmune responses, and toxicity from protein supplements or anabolic agents [13-15]. Our patient had a positive history of testosterone injections and high-protein supplements, which may compound mitochondrial or endothelial stress and exacerbate peripheral nerve damage. In cases of SFN with suspected autoimmune causality, immunomodulatory therapy may be considered. Previous data suggest that intravenous immunoglobulin (IVIG) can be both safe and effective in rigorously selected patients with apparently autoimmune small fiber polyneuropathy. One study demonstrated that IVIG improved both sensory symptoms and autonomic dysfunction, providing a rationale for future prospective trials and highlighting the autoimmune underpinnings of certain SFN presentations [16]. Similarly, systemic corticosteroids have shown therapeutic benefit in specific subsets of cancer-related neuropathic pain (CR-NP), offering rapid analgesia and improved functional outcomes. This suggests that corticosteroids may hold promise for tailored interventions in neuropathic pain syndromes with inflammatory or immune-mediated components, including select cases of post-chemotherapy SFN [17].

The management of SFN remains largely symptomatic and includes lifestyle modification, vitamin optimization, and neuropathic pain agents as needed [3]. Cognitive impairment may improve over time, but targeted neurocognitive rehabilitation can be beneficial [18]. Our case is unique as it illustrates SFN without abnormalities on EMG or NCS in a patient who underwent gastrectomy and chemotherapy. Additionally, it documents concurrent “chemobrain” symptoms despite preserved structural brain imaging. The case also highlights the diagnostic value of adjunctive tools such as Sudoscan, TM Flow, and autonomic testing in detecting SFN when conventional investigations yield normal results.

## Conclusions

In oncology survivors with unexplained neuropathic symptoms and cognitive slowing, clinicians should consider small fiber neuropathy and chemotherapy-related neurotoxicity even if routine EMG and MRI findings are normal. Advanced autonomic and sudomotor testing are valuable in confirming SFN. Early recognition can prevent misdiagnosis, improve quality of life, and guide supportive care strategies in cancer recovery.
